# On a Temporary Substitute for Milk in the Feeding of Infants1Read before the Buffalo Pathological Society, July 18, 1890.

**Published:** 1890-09

**Authors:** DeLancey Rochester

**Affiliations:** 469 Franklin Street


### ON A TEMPORARY SUBSTITUTE FOR MILK IN THE FEEDING OF INFANTS.[1]

By DeLANCEY ROCHESTER, M. D.

Early in March, 1890, an attempt was made to wean a girl baby six months old, because its mother's milk was insufficient. The baby objected seriously to the bottle, nor could she be induced to take food by spoon. The foods tried were sterilized milk (Meigs's Mixture), malted milk, and Carnrick's food. So determined was the objection on the part of the baby to artificial feeding, that she cried and fretted so much at each feeding time as to cause a considerable rise of temperature and vomiting of everything except breast milk. Her temperature ran as high as 104° F. in the groin. The stools were not very frequent, nor very bad in character.

After all attempts at artificial feeding had proved worse than useless, a wet-nurse was procured and the baby again flourished. After the fever had subsided and the vomiting had ceased, the baby broke out in an eruption that was as similar to the measles eruption as could be imagined. The eruption did not irritate her and disappeared in a few days.

About June first the wet-nurse's milk began to give out, and partial artificial feeding was begun, Meigs's milk and cream mixture, sterilized, being the food employed. On this the baby did well.

---

1. Read before the Buffalo Pathological Society, July 18, 1890.

The wet-nurse's milk having given out entirely by the end of a week, the sterilized milk was used alone. For two weeks the baby gained in every way — weight, firmness of flesh, quiet sleep, and happy waking hours. Then the stools began to show curds; but as in every other respect the baby was doing well, nothing was done for this. The last day of June and first of July were excessively hot days, and the baby wilted completely — feverish, restless, fretting, and finally vomiting. Stools remaining as they had been for about a week previous, of good color, but containing curds.

By the advice of Dr. Hopkins she was given sodium bromide to quiet her restlessness, and cool baths to reduce her fever. Nevertheless, she vomited every time that milk was given to her. Finally, she was given toast water and white of egg for twelve hours, with no vomiting. At the end of that time a little malted milk was tried, but she retained it not more than five minutes, when she vomited profusely.

I then began to think what I could give her temporarily as a food until the hyperemia of the stomach should subside and she could again take milk in some form.

White of egg and toast water given cold I knew she could take, so I thought I would look up exactly the composition of egg, and see whether by some addition I could not make a food that would supply all the necessaries for life and growth. In Routh's Infant Feeding and Landois' Physiology I found two analyses of egg — white and yolk — that substantially agreed. I give the percentage composition of yolk of egg, as given by Landois:

| | |
|---|---|
| Water | 51.5 |
| Proteids | 15.0 |
| Fats, etc | 30.0 |
| Mineral matter | 1.4 |
| Pigment extraction | 2.1 |

A glance at this will show that the albuminoids and fats bear about the same relation to each other that they do in human milk. The problem then before us is to dilute the yolk of egg sufficiently with water to make the actual proportions the same as in milk. To do this, of course, it was necessary to obtain the weight of a yolk of egg. I weighed several, and found that each weighed about one ounce — some a little less. Calculating, then, on the total weight of the yolk as one ounce, and remembering that it contained 51.5 per cent. water, to reduce the fifteen parts albuminoids to two parts, it was necessary to add seven ounces of water to the one yolk of egg. The amount of sugar in yolk of egg is very slight, being included in the "etc." added to fats in the above analysis. So, to bring the sugar up to the required amount, it was necessary to add sugar of milk. As there is normally seven per cent. sugar in human milk, this would mean in eight ounces a little over half an ounce. This would make a mixture by weight consisting of:

| | |
|---|---|
| Yolk of egg | 1 oz. |
| Water | 7 oz. |
| Sugar of milk | ½ oz. |

For the convenience of the household I always make these proportions by volume, so I measured the half ounce of sugar of milk and found that it filled six teaspoons in an ordinary medicine glass, so the following formula was prepared:

| | |
|---|---|
| Yolk of egg | No. 1 |
| Sugar of milk | 6 teaspoonfuls. |
| Filtered water | 7 oz. |

Dissolve the sugar of milk in the water and add gradually to the yolk of egg, stirring constantly.

This was fed perfectly cold, in small quantities at a time, for twelve hours, gradually increasing the amount and lengthening the intervals, until finally the full amount was given four times in the twenty-four hours. I once tried warming it slightly — not enough to coagulate the albumen — but the baby objected to it warm, so that was not tried again.

The baby retained this food from the first. After I began with this food it was no longer necessary to give the sodium bromide, but the baths were kept up, three or four being given daily. She was dressed very lightly and made as cool as possible. Luckily the weather also changed and we had three or four cool days. For sixty hours she lived on the egg food alone. During this time the fever left her entirely, and she again broke out in a copious rash, as much like measles as the previous one. At the end of the sixty hours, as she began to show dislike for the egg food, I tried again the malted milk. She retained it and seemed very fond of it. So it was gradually substituted for the egg food, and for the last eight or ten days the baby has lived on malted milk alone, and thrives on it.

The peculiarity of this case is that the stomach was the seat of the chief trouble, the intestines being little, if at all, involved. The peculiarity of the rash, and the great extent of surface that it covered without causing any irritation, is also deserving of mention. The advantages of this egg food, that has been suggested, are that it contains all the necessary ingredients for life and growth in the proportions in which they exist in human milk, although in somewhat different form ; that it must be given cold, and that it is slighly laxative.

I say that it is an advantage that it must be given cold, as I think that it thus has a beneficial action in overcoming the hyperemia of the stomach, and thus prevents further inflammatory developments. It is an advantage that it is slightly laxative, for it thus aids in the discharge of any milk curds that might be in the bowel ready to set up irritative action. This is really a great advantage, for in the congested state of the stomach this baby was unable to retain any laxative medicine, even calomel in small doses being rejected.

469 Franklin Street.

